# Genome-wide analysis of methylation in giant pandas with cataract by methylation-dependent restriction-site associated DNA sequencing (MethylRAD)

**DOI:** 10.1371/journal.pone.0222292

**Published:** 2019-09-25

**Authors:** Yuyan You, Chao Bai, Xuefeng Liu, Maohua Xia, Ting Jia, Xiaoguang Li, Chenglin Zhang, Yucun Chen, Sufen Zhao, Liqin Wang, Wei Wang, Yanqiang Yin, Yunfang Xiu, Lili Niu, Jun Zhou, Tao Ma, Yang Du, Yanhui Liu

**Affiliations:** 1 Beijing Key Laboratory of Captive Wildlife Technologies, Beijing Zoo, Beijing, China; 2 Beijing Zoo, Beijing, China; 3 Strait (Fuzhou) Giant Panda Research and Exchange Centers, Fuzhou, China; 4 Chengdu Zoo, Chengdu, China; 5 Chongqing Zoo, Chongqing, China; New England Biolabs Inc, UNITED STATES

## Abstract

The giant panda *(Ailuropoda melanoleuca*) is a native species to China. They are rare and endangered and are regarded as the ‘national treasure’ and ‘living fossil’ in China. For the time being, there are only about 2500 giant pandas in the world. Therefore, we still have to do much more efforts to protect the giant pandas. In captive wildlife, the cataract incidence of mammalian always increases with age. Currently, in China, the proportion of elderly giant pandas who suffering from cataract has reached 20%. The eye disorder thus has a strong influence on the physical health and life quality of the elderly giant pandas. To discover the genes associated with the pathogenesis of cataract in the elderly giant panda and achieve the goal of early assessment and diagnosis of cataract in giant pandas during aging, we performed whole genome methylation sequencing in 3 giant pandas with cataract and 3 healthy giant pandas using methylation-dependent restriction-site associated DNA sequencing (MethylRAD). In the present study, we obtained 3.62M reads, on average, for each sample, and identified 116 and 242 differentially methylated genes (DMGs) between the two groups under the context of CCGG and CCWGG on genome, respectively. Further KEGG and GO enrichment analyses determined a total of 110 DMGs that are involved in the biological functions associated with pathogenesis of cataract. Among them, 6 DMGs including *EEA1*, *GARS*, *SLITRK4*, *GSTM3*, *CASP3*, and *EGLN3* have been linked with cataract in old age.

## Introduction

Nowadays, a growing number of wild animals have been successfully placed in Zoo. Although the captive animals in Zoo live longer than those in the wild, the aged captive animals (e.g., Malayan Tapir) always encounter various age-related diseases including cataract. Cataract, characterized by the opacification of eye lens, is the most common cause for the blindness of almost all mammals, such as dogs, rhesus monkeys, and humans[1–3]. In addition, increasing age is considered to be the most important risk factor for cataract and a considerable number of cataract are classified as age-related cataract[4]. The loss of vision caused by age-related cataract has great influences on the health status of aged animals. As showed by one previous investigation on the captive rhesus monkeys, cataract attacked 20% of the rhesus monkeys at age of 20–22 years and the rate was still increasing after 26 years of age [1]. In addition, the giant panda (*Ailuropoda melanoleuca*), a world’s most protected rare animals, is also attacked by cataract with age. The studies have shown that the average life span of wild giant pandas is about 15–20 years old, while those in captivity usually live longer and can reach to the age of about 25–30 years[5–6]. Generally, the lifespan of human is 4–4.5-fold longer than the giant panda. The giant panda at the age of 20 years approximately equals human at age of 80–90 years and those aged after 18 years are always served to be aged giant pandas. According to the national survey of eye diseases in aged giant pandas, conducted by Beijing Zoo in 2013, approximately 20% of the aged giant pandas suffered from cataract. Since the giant panda is still an endangered species, the protection of aged giant pandas from cataract has great significances.

Hitherto, a growing number of evidence has shown that genetic factors have large influences on the severity of cataract and play important roles in the development of cataract[7]. For instance, oxidative stress and DNA damage are two common contributors to the many changes in development of age-related cataract[8–10]. Abundant evidence has revealed that genes related to these activities (i.e., oxidative stress and DNA damage) play an important role in the pathogenesis of age-related cataract, such as *SOD1*, *PRDX6*, and *CRYBA4*[11–13]. Epigenetic modifications (e.g., DNA methylation, histone modifications, and non-coding RNA) refer to the alteration of gene activity without any changes in genomic sequence[14–15]. Currently, alteration in epigenetic patterns, in especial DNA methylation, has been closely linked with the cataractogenesis[16]. For example, a reduction of *OGG1* and *CRYAA* expression caused by hypermethylation was observed in lens of eyes with age-related cataract[17–18]. All the existing evidence indicates that the abnormal DNA methylation changes have great contributions to the development of age-related cataract in giant pandas.

In this present study, we performed genome-wide DNA methylation analysis on 3 giant pandas with cataract and 3 healthy giant pandas by methylation-dependent restriction-site associated DNA sequencing (MethylRAD)[19]. Comparison of methylation patterns between the two groups led to the identification of a number of the differentially methylated genes (DMGs) according to the methylation level of CCGG/CCWGG sites. Further analyses showed that the DMGs are preferential located on KEGG pathways and GO terms that have close associations with cataract development. Among these DMGs, some genes (e.g., *CASP3*, *HMGB1*, *EEA1*, and *GARS*) indeed have been proved to be functioning in the pathogenesis of age-related cataract. Taken together, our study illustrates the epigenetic basis of cataract development in giant panda and identifies potential targets for drug intervention in the therapy of age-related cataract. This research work will facilitate the development of precision medical measures for cataract specific to giant pandas.

## Materials and methods

### Sampling and MethylRAD sequencing

The peripheral blood samples were collected from 6 female giant pandas, consisting of 3 cases with cataract and 3 healthy controls. A total of 2 ml blood was draw for each sample during the daily physical examination (without anesthetic). The genomic DNA of blood samples was extracted using phenol-chloroform method (EMD Millipore-516726, Sigma-Aldrich). Blood samples were initially stored at -80°C. Construction of the MethylRAD library has been described by Wang *et al*.[19]. 3 μg genomic DNA for each sample was mixed with FspEI (5U/μl) by a volume ratio of 1:0.8. Then, 30 × Enzyme activator was added to the mixture for digestion reaction, with a volume ratio on 0.5:1. After ligation of adaptor, the product was enriched and purified, and then amplified with PCR reactions. PCR product was further purified using QIAquick PCR Purification Kit. Finally, the short DNA fragments in each library were sequenced on Illumina HiSeq platform by the mode of single-end, 50-bp (Illumina Inc., USA).

### Quality control and reads alignment

To get the clean reads with high quality, we filtered out the poor-quality reads using the threshold of over 15% of bases in a read with quality value of less than 30. In addition, we also removed those reads with a percentage of N greater than 8%[18]. Then, the reads with enzyme sites (enzyme reads) were extracted for subsequent analyses.

The reference genome (AilMel 1.0) of giant panda was downloaded from the National Center for Biotechnology Information (NCBI) with the website: ftp://ftp.ncbi.nlm.nih.gov/genomes/all/GCF/000/004/335/GCF_000004335.2_AilMel_1.0/GCF_000004335.2_AilMel_1.0_genomic.fna.gz). Then, we mapped the enzyme reads to the reference genome of AilMel 1.0 using SOAP version 2.21 (http://soap.genomics.org.cn/) with the parameters: -M 4 -v2 -r 0[20].

### Quantitation and compare of methylation level between two groups

Since the consistency of amplification efficiency for the sequences with equal length, the methylation level of the sites (CCGG/CCWGG) can be quantified by the sequencing depth of the methylation tag. For the MethylRAD-sequencing, the methylation level of each site (CCGG/CCWGG) was represented by RPM (reads per million) as the following formula[21]:

$$RPM=\frac{site\;coverage\;reads\;number*1,000,000}{library\;high\;quality\;reads\;number}$$ (1)

In addition, the methylation level of one certain genic region including upstream/downstream 2000 bp of TSS (transcription start site), gene body, and upstream/downstream 2000 bp of TTS (transcription termination site) was calculated by the sum value of all the methylated sites that are located in the corresponding the region. The methylation data were then analyzed to identify the differentially methylated sites/genes (DMS/Gs) between the case and control groups using the edgeR Bioconductor package that is relied on the number of coverage reads on the sites or genes[22]. Here, the sites/genes with at least 3 reads coverage across the samples in at least one group were retained and those meeting the threshold of p value ≤ 0.05 and |log2FC| > 1 were determined as DMS/Gs, with hypermethylated and hypomethylated.

### Gene annotation and enrichment analysis

The gene annotation information of giant panda was also downloaded from the National Center for Biotechnology Information (NCBI) with the website: ftp://ftp.ncbi.nlm.nih.gov/genomes/all/GCF/000/004/335/GCF_000004335.2_AilMel_1.0/GCF_000004335.2_AilMel_1.0_genomic.gff.gz. The UTR regions of the genes were calculated by using SnpEff tool based on the annotation information[23]. The distributions of the methylated sites on various genomic sequences were calculated by BEDTools[24]. To perform gene set enrichment analysis, we obtained the available gene information of pathways and biological functions from databases of Kyoto Encyclopedia of Genes and Genomes (KEGG), Gene Ontology (GO) and The Comparative Toxicogenomics Database (CDT)[25–27]. We utilized the hypergeometric test to calculate the statistical significance of genes enriched on each biological function.

## Results

### Source of the study samples

In 2013, we looked into 55 old giant pandas in Chinese Zoo, among which 11 (8 females and 3 males) were suffering from cataract. Most of the sufferers were female and over 20 years old. Here, we obtained the genomic DNA samples of peripheral blood cells from 3 female giant pandas with cataract, 2 healthy female giant pandas and 1 male giant panda to perform subsequent genome-wide methylation study. As presented in Table 1, we numbered the 6 giant panda samples as YY-Y, BD-Y, YY-XK, LL-D, JN-D, and XX-XK, respectively. Among these samples, the YY-Y and BD-Y, were healthy ones with an age of approximately 20 years old, YY-XK was healthy with an age of 29 years old. LL-D, JN-D, and XX-XK were ill ones with an age of 36, 32, and 25 years old, respectively. LL-D was died in 2018.

**Table 1:** Basic characteristics of giant pandas.

Name	Spectrum number	Number	Birth year	Status	Sex	Remarks
**YAER**	493	YY-X	1999	Health	Female	-
**BINGDIAN**	520	BD-X	2000	Health	Male	-
**YAYA**	362	YY-XK	1990	Health	Female	-
**LELE**	320	LL-D	1986	Age-related cataract	Female	DEATH
**JINI**	403	JN-D	1993	Age-related cataract	Female	-
**XINXING**	253	XX-XK	1982	Age-related cataract	Female	-

### MethylRAD of the samples

The methylomes of the peripheral blood cells from the 6 giant pandas were generated on HiSeq platform using MethylRAD sequencing (see Materials and Methods)[19]. Here, we obtained 3.62 ± 0.17 million raw sequencing reads, on average, for each sample. After removing the reads with low quality and the reads without enzyme sites, we retained about 1.72 million clean enzyme (FspEi) reads that covering CCGG/CCWGG sites in each sample for the subsequent analyses. We mapped the enzyme reads to the reference genome (AilMel 1.0) of giant panda by using SOAP software version 2.21[20]. On average, about 1.31 million clean reads were mapped to the genome for each sample, the mapping ratio is about 76.66% (Table 2). In this study, we determined the reliable methylated sites by a cutoff of read-coverage no less than 3, and obtained 1 million CCGG sites and 0.32 million CCWGG sites, on average, for each sample. The average coverage depth of CCGG and CCWGG sites is 10.3 and 9.01, respectively (Table 3). Therefore, the sequencing reads satisfy the condition of following analyses.

**Table 2:** Sequencing statistics.

Sample	Raw_Reads	Enzyme_Reads	Mapping_Reads	Ratio
**BD-X**	38034466	17569692	9736908	55.42%
**JN-D**	36512489	18484705	15394010	83.28%
**LL-D**	36551012	16310543	12943264	79.36%
**XX-XK**	32619894	18224002	14453161	79.31%
**YY-X**	37128283	15592184	12710888	81.52%
**YY-XK**	36141855	16776455	13599460	81.06%

**Table 3:** Overall methylation site statistics.

Sample	CCGG		CCWGG	
	Site Number	Depth	Site Number	Depth
**LL-D**	868462	8.44	254302	7.79
**JN-D**	1084114	11.71	341173	10.28
**YY-X**	1026671	9.74	346923	8.48
**BD-X**	1036272	11.23	336356	9.39
**XX-XK**	993084	10.18	311897	8.95
**YY-XK**	1037560	10.47	335027	9.17

### Signatures of DNA methylation in the giant pandas

**Fig 1:**
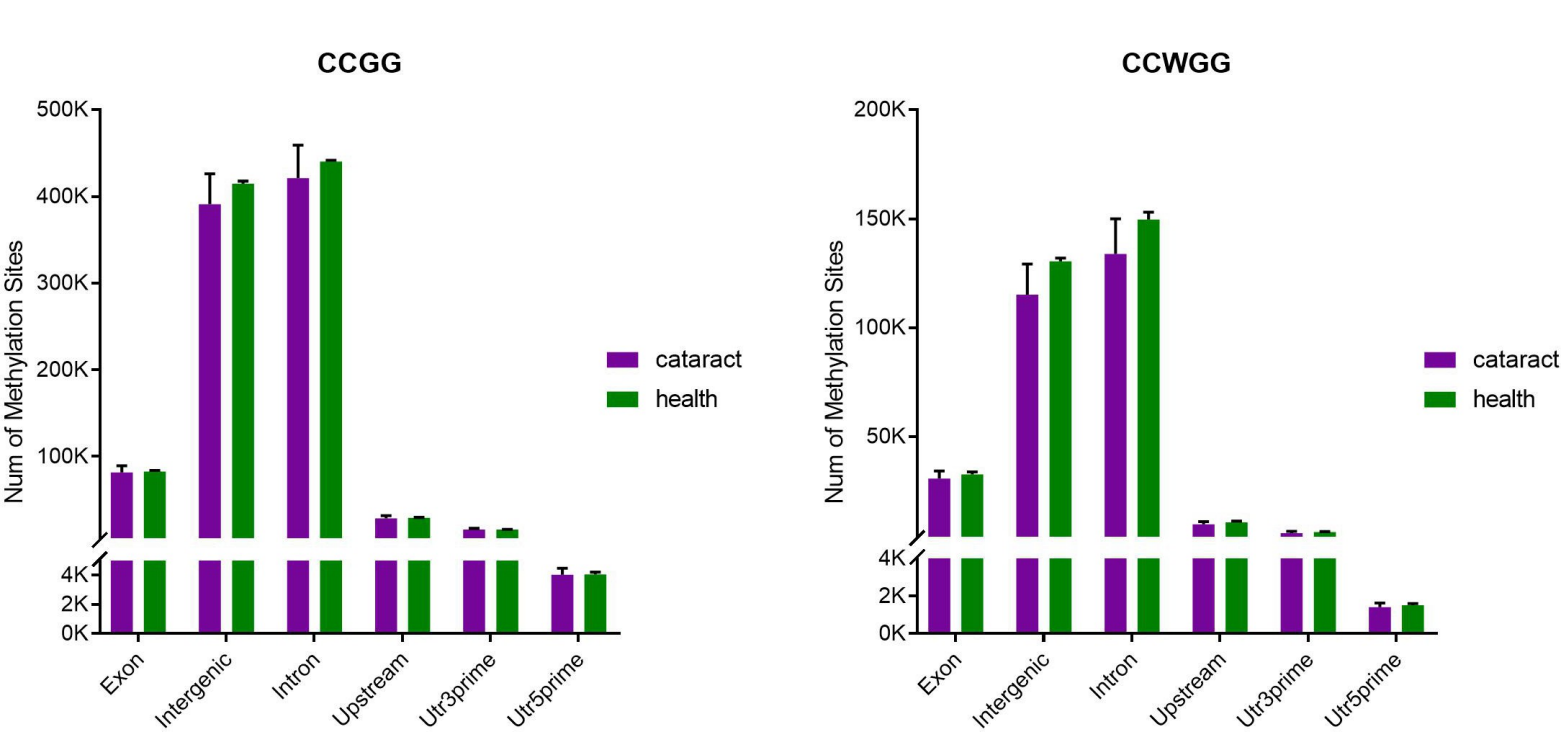
Distribution of methylation sites.

Then, we analyzed the distribution of the methylated CCGG/CCWGG sites on distinct genomic sequences, including Utr3prime, Utr5prime, Upstream, Exon, Intron, and intergenic regions (Fig 1). Among them, the sites were most located in intergenic and intron regions, the next in exon and upstream, and the least in Utr3prime and Utr5prime regions. In addition, results revealed that the distribution of methylated CCGG and CCWGG sites on various genomic regions was very similar in the 3 healthy giant pandas, while a marked difference was observed in the 3 giant panda that suffering from cataract. For instance, the number of methylated CCGG and CCWGG sites located in the various genomic regions was fewest in LL-D, moderate in XX-XK, and largest in JN-D. In addition, we found that the methylated CCGG and CCWGG sites in LL-D and XX-XK were smaller than the healthy giant pandas. However, for the methylated sites in JN-D, the number of CCGG sites was larger than healthy giant pandas, while the number of CCWGG sites was similar with healthy giant pandas.

**Fig 2:**
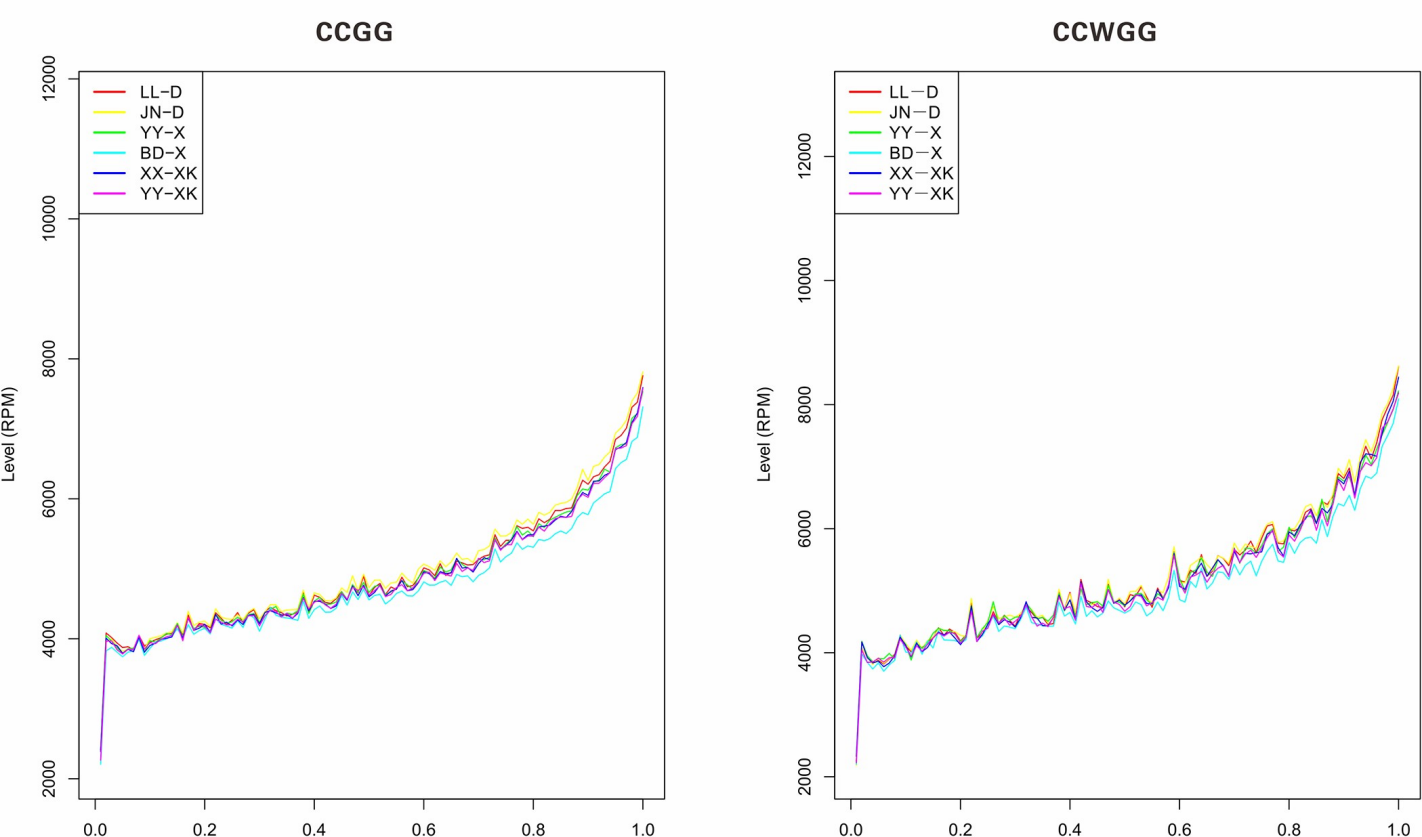
Methylation level of gene region.

In addition, we analyzed the overall methylation pattern of different positions on the genic regions. As presented in Fig 2 and S1 Table, the methylation level based on CCGG and CCWGG sites was gradually rising from the initial position of genic region, occurred a turning point of rising tread, then continued to rise and reach the highest value at the end position. Moreover, no obvious differences were observed in the 6 giant pandas.

### Genes differentially methylated in the case and control groups

**Fig 3:**
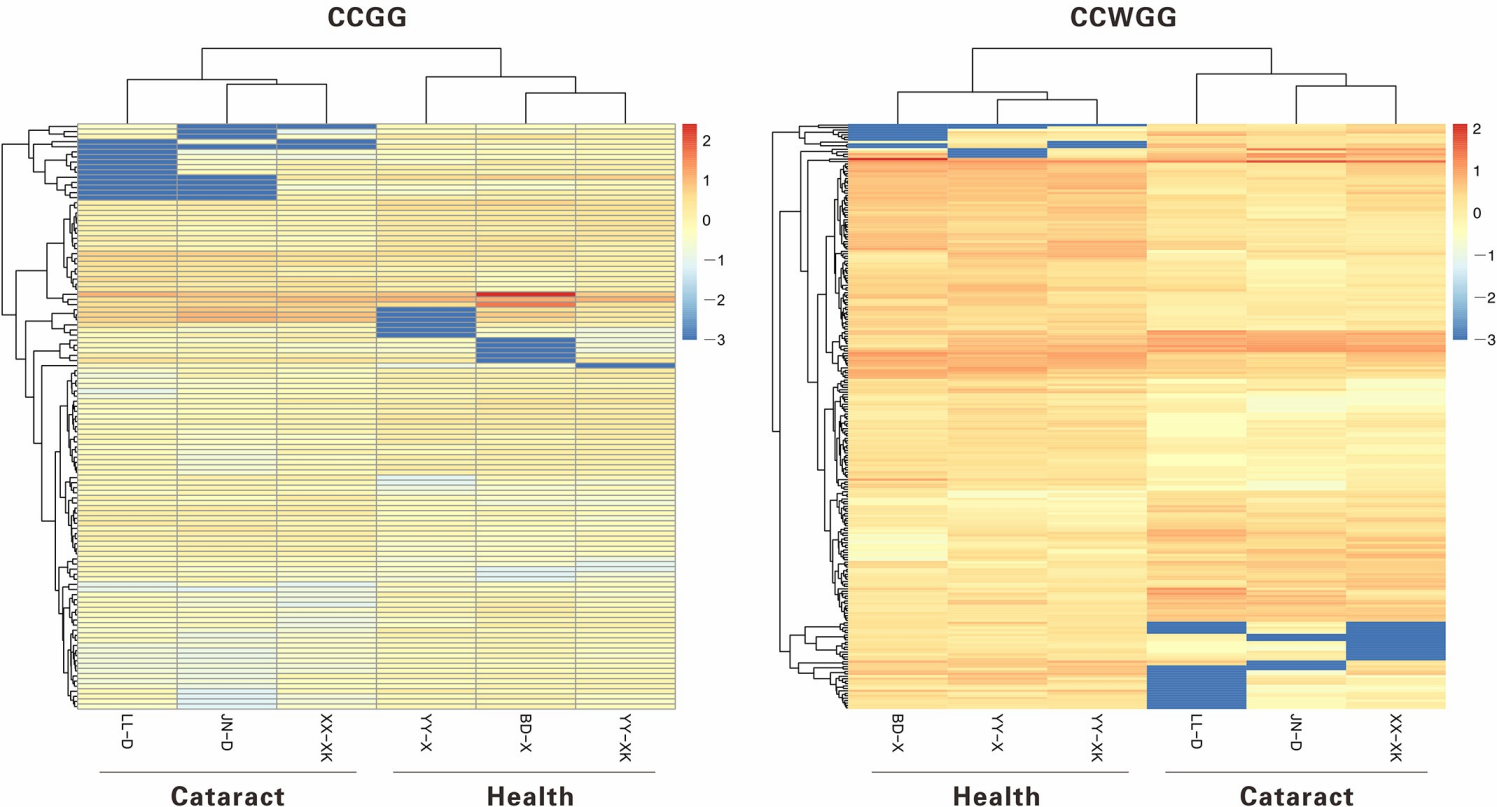
Cluster heat map of differentially methylated genes between group.

**Fig 4:**
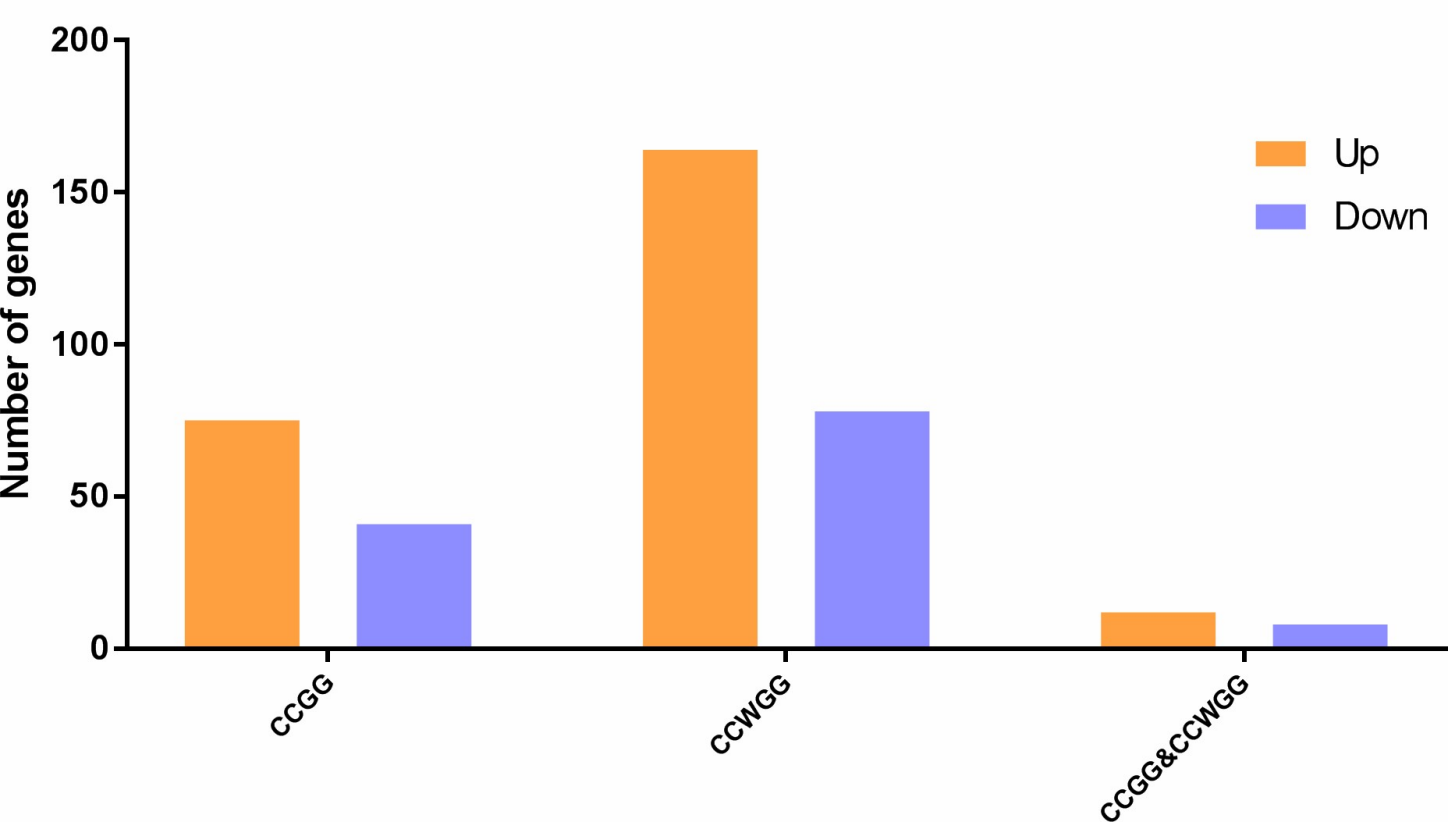
Gene statistics of different methylation levels.

In the present study, we calculated the methylation level of genes based on the CCGG and CCWGG sites, respectively, and identified the DMGs between the case and control groups using a threshold of *P* value ≤ 0.05 and absolute log2FC value > 1 (see Materials and Methods). Here, we identified a total of 116 DMGs by the CCGG sites, including 75 hypermethylated genes and 41 hypomethylated genes. Moreover, we determined 242 DMGs by the CCWGG sites, including 164 hypermethylated genes and 78 hypomethylated genes. The heatmap plot showed a different methylation pattern between the two groups, with genes highly methylated in patients presenting low methylation level in the healthy samples and vice versa. For both the CCWGG and CCGG sites, there were a large number of genes with hypermethylation in healthy group (Fig 3). Among them, there were 20 DMGs that were identified by both the CCGG and CCWGG sites, containing 12 hypermethylated genes and 8 hypomethylated genes (Fig 4 and S2 Table).

### Pathway-level functions of the DMGs in the development of cataract

**Fig 5:**
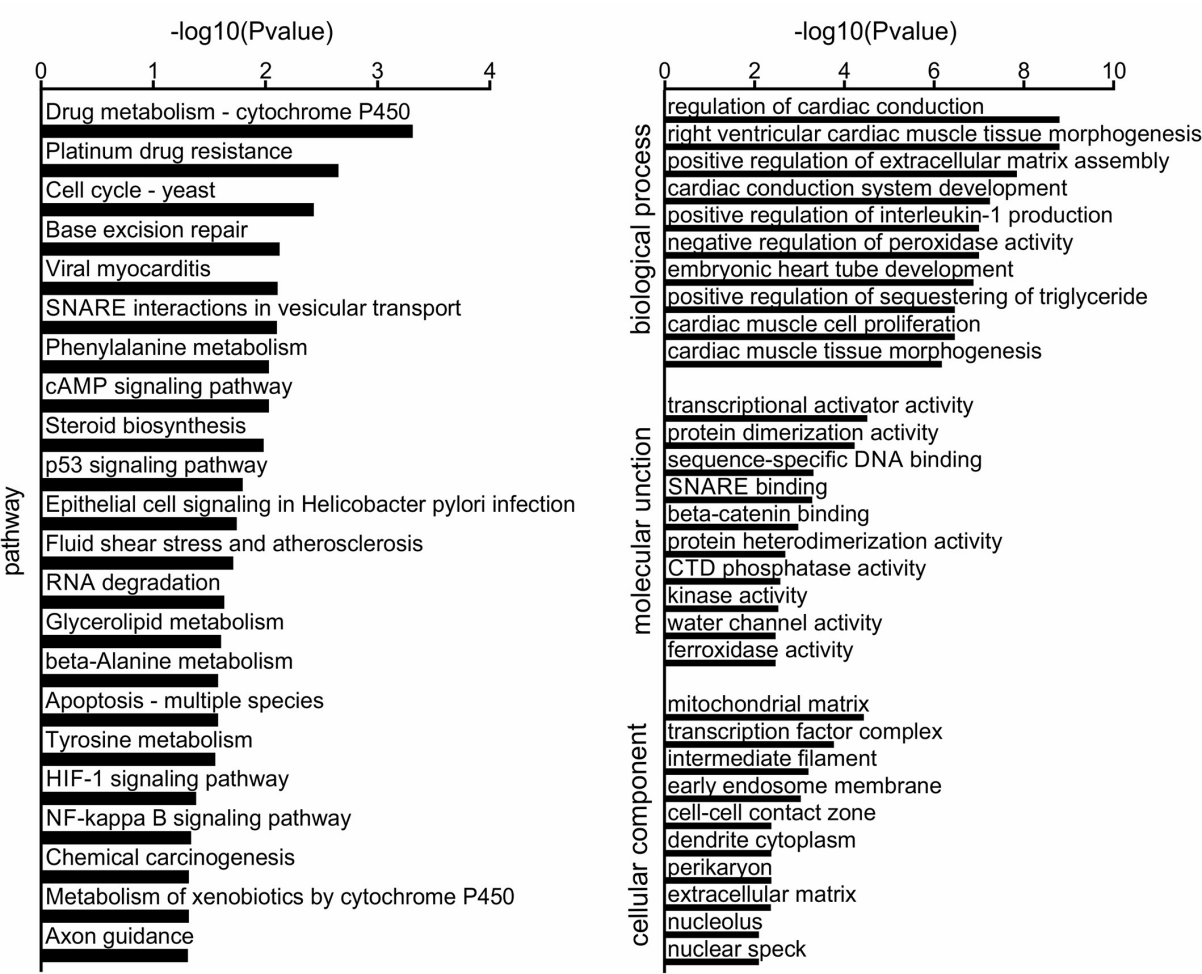
Functional enrichment of genes related to different methylation levels.

To determine the signaling pathways potentially associated with age-related cataract, we first performed KEGG enrichment analysis and searched the available annotation information from CTD database[25, 27]. By using a threshold of *P* value < 0.05, we identified 15 and 55 enriched KEGG signaling pathways for the CCGG- and CCWGG-based DMGs, respectively. Among them, the CCGG-based signaling pathways contained 27 DMGs, including 22 hypermethylated genes and 5 hypomethylated genes; while the CCWGG-based signaling pathways contained 96 DMGs, including 80 hypermethylated genes and 16 hypomethylated genes. In addition, there were 3 enriched pathways according to both CCGG- and CCWGG-based DMGs. A total of 10 DMGs were located in these 3 signaling pathways, including 8 hypermethylated genes and 2 hypomethylated genes. We then summarized the findings of KEGG signaling pathways to assess their associations with cataract pathogenesis. The pathways associated with genetic information processing included base excision repair (cataract-related genes: *HMGB1*, hypomethylated in aged giant pandas with cataract), SNARE interactions in vesicular transport (*STX19*, hypermethylated), and RNA degradation (*MPHOSPH6* and *TTC37*, both were hypermethylated). The pathways related with environmental information processing contained NF-kappa B signaling pathway (*CCL19*, hypomethylated), cAMP signaling pathway, HIF-1 signaling pathway (*LOC100484901*, *PDK1*, and *EGLN3*, all were hypermethylated). On the level of cellular process, there were 5 pathways, 3 of which had some associations with cataract, including cell cycle (*ORC6* and *CDC7*, both were hypermethylated), apoptosis (*CASP3*, hypermethylated), p53 signaling pathway (*TP53I3* and *CASP3*, both were hypermethylated). On the level of metabolism, there were 17 pathways, 7 of which were related with cataract, including drug metabolism-cytochrome P450 (*FMO5* and *GSTM3*, both were hypermethylated), glycerolipid metabolism (*LPL*, hypermethylated), beta-Alanine metabolism (*LOC100474209*, hypermethylated), tyrosine metabolism (*LOC100474209*, hypermethylated), phenylalanine metabolism (*LOC100474209*, hypermethylated), metabolism of xenobiotics by cytochrome P450 (*GSTM3*, hypermethylated), steroid biosynthesis (*MSMO1*, hypermethylated). For organismal systems, there were 16 pathways, 1 of which was related with cataract (i.e., axon guidance). For the level of human disease, there were 19 pathways, 5 of which were associated with cataract. Those were Epithelial cell signaling in Helicobacter pylori infection (*ATP6V1C1* and *CASP3*, both were hypermethylated), Platinum drug resistance (*TOP2B*, *GSTM3*, and *CASP3*, all were hypermethylated), Viral myocarditis (*LOC100463889* and *CASP3*, both were hypermethylated), Fluid shear stress and atherosclerosis (*LOC100484313*, *GSTM3*, and *ACVR2A*, all were hypermethylated), Chemical carcinogenesis (*GSTM3*, hypermethylated) (Fig 5, Table 4 and S3 Table).

**Table 4:** Kegg enrichment of genes related to different methylation levels.

Class	Pathway	CCGG_pval	CCWGG_pval
**Genetic information processing**	Base excision repair	0.007442542	NA
	SNARE interactions in vesicular transport	0.007902734	NA
	RNA degradation	NA	0.023225782
**Environmental information processing**	NF-kappa B signaling pathway	0.045908268	NA
	cAMP signaling pathway	0.043841452	0.211954698
	HIF-1 signaling pathway	NA	0.041378905
**Cellular processes**	Cell cycle—yeast	0.032936174	0.111987176
	Apoptosis—multiple species	NA	0.026345221
	p53 signaling pathway	NA	0.015908608
**Metabolism**	Drug metabolism—cytochrome P450	0.011470212	0.042177597
	Glycerolipid metabolism	0.024752064	NA
	beta-Alanine metabolism	NA	0.026345221
	Steroid biosynthesis	NA	0.010314036
	Tyrosine metabolism	NA	0.027955927
	Phenylalanine metabolism	NA	0.009276792
	Metabolism of xenobiotics by cytochrome P450	NA	0.048077893
**Organismal systems**	Axon guidance	NA	0.04905591
**Human diseases**	Epithelial cell signaling in Helicobacter pylori infection	NA	0.017941728
	Platinum drug resistance	NA	0.002231982
	Viral myocarditis	NA	0.00778076
	Chemical carcinogenesis	NA	0.048077893
	Fluid shear stress and atherosclerosis	NA	0.019346472

### GO-level functions of the DMGs in cataract

Similarly, we also conducted GO enrichment analysis for the DMGs and then found the cataract-related GO term by CTD database[26–27]. The CCGG-based DMGs were enriched in 396 GO terms, containing 59 DMGs (36 hypermethylated genes, 23 hypomethylated genes). The CCWGG-based DMGs were enriched in 681 GO terms, including 129 DMGs (88 hypermethylated genes and 41 hypomethylated genes). There were 105 GO terms that were identified with both CCGG- and CCWGG-based DMGs. These terms contained 59 DMGs, among which 7 genes carried both differentially methylated CCGG and CCWGG sites. Under the cellular component, there were 102 enriched terms, among which 26 terms had an association with cataract. In addition, there were 13 terms containing DMGs with same directional methylation changes, such as intermediate filament (*LOC105240942* and *LOC100465932*, hypermethylated), membrane raft (*RRK2*, *LOC100476759*, and *GNAI1*, hypermethylated), midbody (*KIF20B*, *GNAI1*, and *ASPM*, hypermethylated). For molecular function, the DMGs were enriched in 181 terms, among which 29 were associated with cataract. 16 of the 29 terms had DMGs with same directional changes in one same term, such as structural constituent of cytoskeleton (*LOC100473181* and *TUBD1*, hypermethylated), SNARE binding (*STX19* and *LRRK2*, hypermethylated), beta-catenin binding (*SOX9* and *SOX17*, hypomethylated), calmodulin binding (*EEA1* and *MIP*, hypermethylated). For biological process, the DMGs were enriched in 690 terms, 219 of which were linked with cataract. 182 of the 219 terms had DMGs showing same directional changes, such as cardiac muscle tissue development (*NKX2-5*, hypermethylated), BMP signaling pathway (*ACVR2A*, *TMEM100*, and *NKX2-5*, hypermethylated), cellular metabolic process (*PDP1* and *PDK1*, hypermethylated), cholesterol metabolic process (*PCTP* and *LOC100476613*, hypomethylated), and regulation of membrane potential (*GABRG1*, *LRRK2*, and *LOC105234775*, hypermethylated) (Fig 5, Table 5 and S4 Table).

**Table 5:** GO enrichment of genes related to different methylation levels.

CClass	GO_id	GO_def	CCGG_pval	CCWGG_pval
**Cellular_component**	GO:0005882	intermediate filament	0.026127779	0.02394968
	GO:0005667	transcription factor complex	0.016392771	0.010378895
	GO:0016021	integral component of membrane	0.009779408	0.909683214
	GO:0016607	nuclear speck	0.032553867	0.245809679
	GO:0005623	cell	0.018123614	NA
	GO:0005615	extracellular space	0.03795066	0.376522069
	GO:0030018	Z disc	0.049478525	0.218824299
	GO:0031012	extracellular matrix	0.030158622	0.14389526
	GO:0016363	nuclear matrix	0.030158622	NA
	GO:0031901	early endosome membrane	0.0046239	0.200585267
	GO:0032839	dendrite cytoplasm	NA	0.004216355
	GO:0043204	perikaryon	NA	0.004242455
	GO:0030426	growth cone	NA	0.044360918
	GO:0000781	chromosome, telomeric region	NA	0.02360783
	GO:0043005	neuron projection	NA	0.040184316
	GO:0045121	membrane raft	NA	0.025764856
	GO:0030666	endocytic vesicle membrane	NA	0.029446371
	GO:0044291	cell-cell contact zone	NA	0.004216355
	GO:0030496	midbody	NA	0.025032919
	GO:0005730	nucleolus	0.269129545	0.029526994
	GO:0005759	mitochondrial matrix	NA	3.68158E-05
	GO:0005921	gap junction	NA	0.008306393
	GO:0005637	nuclear inner membrane	NA	0.038017218
	GO:0042645	mitochondrial nucleoid	NA	0.044990081
	GO:0051233	spindle midzone	NA	0.010811581
	GO:0031410	cytoplasmic vesicle	NA	0.024313727
**Molecular_function**	GO:0043565	sequence-specific DNA binding	0.010499085	0.046297174
	GO:0046983	protein dimerization activity	0.00322677	0.018315105
	GO:0001228	transcriptional activator activity, RNA polymerase II transcription regulatory region sequence-specific binding	0.001586706	0.019205381
	GO:0003690	double-stranded DNA binding	0.018123614	NA
	GO:0005200	structural constituent of cytoskeleton	0.028518085	0.137070187
	GO:0000149	SNARE binding	0.00981639	0.052393303
	GO:0003700	transcription factor activity, sequence-specific DNA binding	0.043134111	0.610799417
	GO:0003735	structural constituent of ribosome	0.043990433	0.305199845
	GO:0046982	protein heterodimerization activity	0.015107415	0.135882043
	GO:0004129	cytochrome-c oxidase activity	0.003867281	NA
	GO:0001077	transcriptional activator activity, RNA polymerase II core promoter proximal region sequence-specific binding	0.022134047	0.43540707
	GO:0003682	chromatin binding	0.02893346	0.547748796
	GO:0008013	beta-catenin binding	0.014292521	0.074006113
	GO:0003756	protein disulfide isomerase activity	NA	0.010811581
	GO:0016740	transferase activity	NA	0.02360783
	GO:0005506	iron ion binding	NA	0.025764856
	GO:0031418	L-ascorbic acid binding	NA	0.015108679
	GO:0016301	kinase activity	NA	0.002936508
	GO:0004322	ferroxidase activity	NA	0.003395245
	GO:0005516	calmodulin binding	NA	0.008959942
	GO:0005215	transporter activity	NA	0.012998915
	GO:0000254	C-4 methylsterol oxidase activity	NA	0
	GO:0051059	NF-kappaB binding	NA	0.008306393
	GO:0004364	glutathione transferase activity	NA	0.020016375
	GO:0008420	CTD phosphatase activity	NA	0.00265811
	GO:0004672	protein kinase activity	0.080285293	0.028820813
	GO:0015250	water channel activity	NA	0.003395245
	GO:0005212	structural constituent of eye lens	NA	0.018315105
	GO:0000166	nucleotide binding	NA	0.019035545
**Biological_process**	GO:0048738	cardiac muscle tissue development	0.000445093	0.00265811
	GO:0055007	cardiac muscle cell differentiation	0.004207877	0.02360783
	GO:0007283	spermatogenesis	0.00351856	0.040669737
	GO:0001570	vasculogenesis	0.0103349	0.000539043
	GO:0050821	protein stabilization	0.040750902	0.044360918
	GO:0090090	negative regulation of canonical Wnt signaling pathway	0.004781429	0.000191203
	GO:0060047	heart contraction	0.000867457	0.005119712
	GO:0060038	cardiac muscle cell proliferation	0.000239694	0.001442798
	GO:0030509	BMP signaling pathway	0.012527107	0.000792502
	GO:0003007	heart morphogenesis	0.003867281	0.021781089
	GO:0003161	cardiac conduction system development	9.63811E-05	0.000584755
	GO:0031295	T cell costimulation	0.003867281	0.001486876
	GO:0055008	cardiac muscle tissue morphogenesis	0.000334694	0.002006702
	GO:0043491	protein kinase B signaling	0.002369396	0.013606467
	GO:0045860	positive regulation of protein kinase activity	0.006961412	0.038017218
	GO:0070328	triglyceride homeostasis	0.001638839	0.009521989
	GO:0035050	embryonic heart tube development	0.000867457	0.000151247
	GO:1901203	positive regulation of extracellular matrix assembly	4.83169E-05	0.000294308
	GO:0060048	cardiac muscle contraction	0.005309692	0.029446371
	GO:0003221	right ventricular cardiac muscle tissue morphogenesis	1.61479E-05	9.87507E-05
	GO:0034504	protein localization to nucleus	0.00322677	0.018315105
	GO:0010890	positive regulation of sequestering of triglyceride	0.000239694	0.001442798
	GO:1903779	regulation of cardiac conduction	1.61479E-05	9.87507E-05
	GO:0007017	microtubule-based process	0.005703272	0.031507479
	GO:0046330	positive regulation of JNK cascade	0.008333403	0.044990081
	GO:0008284	positive regulation of cell proliferation	0.014066279	0.402952797
	GO:0030857	negative regulation of epithelial cell differentiation	0.000160215	NA
	GO:0014068	positive regulation of phosphatidylinositol 3-kinase signaling	0.000105939	NA
	GO:0006954	inflammatory response	0.001597038	0.158092846
	GO:0043065	positive regulation of apoptotic process	0.025787421	NA
	GO:0030154	cell differentiation	0.000966605	0.193072503
	GO:0001934	positive regulation of protein phosphorylation	0.033549391	0.157750125
	GO:0007507	heart development	0.006927629	0.071080057
	GO:0009408	response to heat	0.003867281	NA
	GO:0010458	exit from mitosis	0.000334694	NA
	GO:0010628	positive regulation of gene expression	0.012153509	0.113861252
	GO:0014032	neural crest cell development	0.000334694	NA
	GO:0030903	notochord development	0.000160215	NA
	GO:0070830	bicellular tight junction assembly	0.00652924	NA
	GO:0051897	positive regulation of protein kinase B signaling	0.017457942	NA
	GO:0060009	Sertoli cell development	0.000334694	NA
	GO:0070374	positive regulation of ERK1 and ERK2 cascade	0.038899676	NA
	GO:0031532	actin cytoskeleton reorganization	0.013692701	NA
	GO:0010942	positive regulation of cell death	0.001038232	NA
	GO:0032868	response to insulin	0.004207877	NA
	GO:0001502	cartilage condensation	0.001038232	NA
	GO:0032757	positive regulation of interleukin-8 production	0.001223799	NA
	GO:0090023	positive regulation of neutrophil chemotaxis	0.000867457	NA
	GO:0008584	male gonad development	0.011407572	0.060195631
	GO:0000902	cell morphogenesis	0.013692701	NA
	GO:0050790	regulation of catalytic activity	0.012527107	NA
	GO:0007417	central nervous system development	0.034419441	NA
	GO:0045666	positive regulation of neuron differentiation	0.020184093	0.101044793
	GO:0071560	cellular response to transforming growth factor beta stimulus	0.004561885	NA
	GO:0001894	tissue homeostasis	0.000570767	NA
	GO:0032735	positive regulation of interleukin-12 production	0.000867457	NA
	GO:0050679	positive regulation of epithelial cell proliferation	0.006961412	NA
	GO:0030858	positive regulation of epithelial cell differentiation	9.63811E-05	NA
	GO:0045931	positive regulation of mitotic cell cycle	0.002111633	NA
	GO:0032496	response to lipopolysaccharide	0.026127779	NA
	GO:0014911	positive regulation of smooth muscle cell migration	0.001638839	NA
	GO:0006955	immune response	0.034996371	0.259172857
	GO:0070168	negative regulation of biomineral tissue development	1.61479E-05	NA
	GO:0071260	cellular response to mechanical stimulus	0.007406235	NA
	GO:0007257	activation of JUN kinase activity	0.004929193	NA
	GO:0010629	negative regulation of gene expression	0.04649899	NA
	GO:0030097	hemopoiesis	0.012527107	0.065604136
	GO:0050727	regulation of inflammatory response	0.006109824	NA
	GO:0030335	positive regulation of cell migration	0.049478525	NA
	GO:0032760	positive regulation of tumor necrosis factor production	0.00013679	NA
	GO:0045893	positive regulation of transcription, DNA-templated	0.048018346	0.248256192
	GO:0071364	cellular response to epidermal growth factor stimulus	0.001424041	NA
	GO:0030879	mammary gland development	0.001038232	NA
	GO:0060174	limb bud formation	0.000445093	NA
	GO:0002062	chondrocyte differentiation	0.004207877	NA
	GO:0001501	skeletal system development	0.039821147	NA
	GO:0032755	positive regulation of interleukin-6 production	0.003540208	NA
	GO:0043123	positive regulation of I-kappaB kinase/NF-kappaB signaling	0.000502616	0.062602373
	GO:0032732	positive regulation of interleukin-1 production	0	NA
	GO:0006935	chemotaxis	0.029333711	0.140473622
	GO:0050776	regulation of immune response	0.007406235	NA
	GO:0050718	positive regulation of interleukin-1 beta secretion	0.000711596	NA
	GO:2000020	positive regulation of male gonad development	9.63811E-05	NA
	GO:0032332	positive regulation of chondrocyte differentiation	0.000711596	NA
	GO:0007186	G-protein coupled receptor signaling pathway	0.001350659	0.352837434
	GO:0051216	cartilage development	0.009309902	NA
	GO:0001837	epithelial to mesenchymal transition	0.001638839	NA
	GO:0006915	apoptotic process	0.01819573	0.08355214
	GO:0048469	cell maturation	0.004207877	NA
	GO:0007517	muscle organ development	0.019486784	NA
	GO:0007626	locomotory behavior	0.023824179	NA
	GO:0001503	ossification	0.020184093	NA
	GO:0006338	chromatin remodeling	0.002314778	NA
	GO:0006309	apoptotic DNA fragmentation	0.000570767	NA
	GO:0001541	ovarian follicle development	0.00322677	NA
	GO:0007010	cytoskeleton organization	0.042634951	NA
	GO:0016042	lipid catabolic process	0.031835951	0.150790378
	GO:0010976	positive regulation of neuron projection development	0.018123614	NA
	GO:0071300	cellular response to retinoic acid	0.011961532	NA
	GO:0007595	lactation	0.002111633	NA
	GO:0045807	positive regulation of endocytosis	0.000867457	NA
	GO:0071599	otic vesicle development	0.000160215	NA
	GO:0031175	neuron projection development	0.039821147	0.182544302
	GO:0045732	positive regulation of protein catabolic process	0.014292521	0.074006113
	GO:0032436	positive regulation of proteasomal ubiquitin-dependent protein catabolic process	NA	0.006830072
	GO:0090201	negative regulation of release of cytochrome c from mitochondria	NA	0.004216355
	GO:0044237	cellular metabolic process	NA	0.00213563
	GO:0014044	Schwann cell development	NA	0.000584755
	GO:0051092	positive regulation of NF-kappaB transcription factor activity	NA	0.00607258
	GO:0016310	phosphorylation	NA	0.001486876
	GO:0030316	osteoclast differentiation	NA	0.013606467
	GO:0050848	regulation of calcium-mediated signaling	NA	0.000968205
	GO:0042789	mRNA transcription from RNA polymerase II promoter	NA	0.003395245
	GO:0002376	immune system process	NA	0.020016375
	GO:0051402	neuron apoptotic process	NA	0.001486876
	GO:0070509	calcium ion import	NA	0.02360783
	GO:0070940	dephosphorylation of RNA polymerase II C-terminal domain	NA	0.000968205
	GO:0050999	regulation of nitric-oxide synthase activity	NA	0.004216355
	GO:0032967	positive regulation of collagen biosynthetic process	NA	0.005119712
	GO:0006090	pyruvate metabolic process	NA	0.000494847
	GO:0072593	reactive oxygen species metabolic process	NA	0.02360783
	GO:0071773	cellular response to BMP stimulus	NA	0.015108679
	GO:1900017	positive regulation of cytokine production involved in inflammatory response	NA	0.003395245
	GO:1901800	positive regulation of proteasomal protein catabolic process	NA	0.005119712
	GO:0070997	neuron death	NA	0.005119712
	GO:0018105	peptidyl-serine phosphorylation	NA	0.029270389
	GO:0043525	positive regulation of neuron apoptotic process	NA	0.021781089
	GO:0043537	negative regulation of blood vessel endothelial cell migration	NA	0.002006702
	GO:0030239	myofibril assembly	NA	0.004216355
	GO:0008203	cholesterol metabolic process	NA	0.00545343
	GO:0032092	positive regulation of protein binding	NA	0.003560189
	GO:0001937	negative regulation of endothelial cell proliferation	NA	0.000596502
	GO:0030889	negative regulation of B cell proliferation	NA	0.003395245
	GO:0060914	heart formation	NA	0.001442798
	GO:0007015	actin filament organization	NA	0.010784844
	GO:0021766	hippocampus development	NA	0.042616389
	GO:0006914	autophagy	NA	0.04028559
	GO:0006006	glucose metabolic process	NA	0.003797886
	GO:0090263	positive regulation of canonical Wnt signaling pathway	NA	0.030403443
	GO:0000165	MAPK cascade	0.079101181	0.02803752
	GO:0006853	carnitine shuttle	NA	0.001442798
	GO:0060135	maternal process involved in female pregnancy	NA	0.002006702
	GO:0007080	mitotic metaphase plate congression	NA	0.038017218
	GO:0006635	fatty acid beta-oxidation	NA	0.040291697
	GO:0010718	positive regulation of epithelial to mesenchymal transition	NA	0.012173591
	GO:0008340	determination of adult lifespan	NA	0.002006702
	GO:0048589	developmental growth	NA	0.015108679
	GO:0007005	mitochondrion organization	NA	0.014271521
	GO:0007040	lysosome organization	NA	0.025495204
	GO:0016525	negative regulation of angiogenesis	NA	0.00545343
	GO:0007214	gamma-aminobutyric acid signaling pathway	NA	0.000596502
	GO:0031397	negative regulation of protein ubiquitination	NA	0.025495204
	GO:0008219	cell death	NA	0.018315105
	GO:0007049	cell cycle	NA	0.00800259
	GO:0050877	neurological system process	NA	0.010154547
	GO:0048812	neuron projection morphogenesis	NA	0.004637323
	GO:0021549	cerebellum development	NA	0.029446371
	GO:0042220	response to cocaine	NA	0.005119712
	GO:0042752	regulation of circadian rhythm	NA	0.042616389
	GO:0007519	skeletal muscle tissue development	NA	0.000242571
	GO:0072001	renal system development	NA	0.002006702
	GO:0008016	regulation of heart contraction	NA	0.009521989
	GO:0050896	response to stimulus	NA	0.016635336
	GO:1902476	chloride transmembrane transport	NA	0.02394968
	GO:0006833	water transport	NA	0.001486876
	GO:0042593	glucose homeostasis	NA	0.02394968
	GO:0032091	negative regulation of protein binding	NA	0.040291697
	GO:1903215	negative regulation of protein targeting to mitochondrion	NA	0.000294308
	GO:0001816	cytokine production	NA	0.004216355
	GO:2000484	positive regulation of interleukin-8 secretion	NA	0.000584755
	GO:0045648	positive regulation of erythrocyte differentiation	NA	0.008306393
	GO:0009566	fertilization	NA	0.033623847
	GO:0042713	sperm ejaculation	NA	9.87507E-05
	GO:0008631	intrinsic apoptotic signaling pathway in response to oxidative stress	NA	0.00265811
	GO:0071158	positive regulation of cell cycle arrest	NA	0.006103617
	GO:2000573	positive regulation of DNA biosynthetic process	NA	0.001442798
	GO:0006977	DNA damage response, signal transduction by p53 class mediator resulting in cell cycle arrest	NA	0.027441835
	GO:0006096	glycolytic process	NA	0.031507479
	GO:0021987	cerebral cortex development	NA	0.003239129
	GO:0002244	hematopoietic progenitor cell differentiation	NA	0.018325705
	GO:2000469	negative regulation of peroxidase activity	NA	0
	GO:0016126	sterol biosynthetic process	NA	0.00265811
	GO:0019915	lipid storage	NA	0.016678719
	GO:0030216	keratinocyte differentiation	NA	0.029446371
	GO:0051091	positive regulation of sequence-specific DNA binding transcription factor activity	NA	0.004016009
	GO:0006633	fatty acid biosynthetic process	NA	0.047411578
	GO:0030308	negative regulation of cell growth	NA	0.037679788
	GO:0006357	regulation of transcription from RNA polymerase II promoter	0.102048797	0.021169358
	GO:0046676	negative regulation of insulin secretion	NA	0.018315105
	GO:1902236	negative regulation of endoplasmic reticulum stress-induced intrinsic apoptotic signaling pathway	NA	0.007166394
	GO:0045665	negative regulation of neuron differentiation	NA	0.00733058
	GO:0010906	regulation of glucose metabolic process	NA	0.008306393
	GO:1901215	negative regulation of neuron death	NA	0.000151247
	GO:0031398	positive regulation of protein ubiquitination	NA	0.004258969
	GO:0071346	cellular response to interferon-gamma	NA	0.027441835
	GO:0071850	mitotic cell cycle arrest	NA	7.81376E-05
	GO:0050715	positive regulation of cytokine secretion	NA	0.008306393
	GO:0006956	complement activation	NA	0.000584755
	GO:0045840	positive regulation of mitotic nuclear division	NA	0.006103617
	GO:0048148	behavioral response to cocaine	NA	0.001442798
	GO:0010508	positive regulation of autophagy	NA	0.027441835
	GO:0032355	response to estradiol	NA	0.047411578
	GO:0045597	positive regulation of cell differentiation	NA	0.018315105
	GO:0022038	corpus callosum development	NA	0.000968205
	GO:0051646	mitochondrion localization	NA	0.001442798
	GO:0019722	calcium-mediated signaling	NA	0.047411578
	GO:0035641	locomotory exploration behavior	NA	0.003395245
	GO:0090394	negative regulation of excitatory postsynaptic potential	NA	0.002006702
	GO:0048167	regulation of synaptic plasticity	NA	0.020016375
	GO:0030182	neuron differentiation	NA	0.044360918
	GO:2001214	positive regulation of vasculogenesis	NA	0.00265811
	GO:0043409	negative regulation of MAPK cascade	NA	0.001442798
	GO:1903071	positive regulation of ER-associated ubiquitin-dependent protein catabolic process	NA	0.003395245
	GO:1901741	positive regulation of myoblast fusion	NA	0.005119712
	GO:0006749	glutathione metabolic process	NA	0.029446371
	GO:0048514	blood vessel morphogenesis	NA	0.006103617
	GO:0042391	regulation of membrane potential	NA	0.012998915
	GO:0046855	inositol phosphate dephosphorylation	NA	0.00265811
	GO:1901214	regulation of neuron death	NA	0.006103617

### Roles of the DMGs in cataract

In this study, we identified a total of 338 DMGs between the groups. Among the DMGs, 116 have been previously supposed to be potentially linked with the development of cataract (S5 Table). Base on the results of enrichment analysis, we selected 16 DMGs associated with the cataract-related KEGG pathways and 108 DMGs involved in the cataract-related GO terms. By combing the results of KEGG and GO enrichment analyses, we obtained a total of 110 DMGs (Table 6), among which 6 have been linked with the cataract in old age in previous reports that were *EEA1*, *GARS*, *SLITRK4*, *GSTM3*, *CASP3*, and *EGLN3*.

**Table 6:** Candidate gene.

Gene_ID	Gene_name	CCGG	CCWGG
**Gene20211**	**EEA1**	NA	UP
**Gene8851**	**GARS**	NA	UP
**Gene13601**	**SLITRK4**	Down	NA
**Gene8796**	**GSTM3**	NA	UP
**Gene2171**	**CASP3**	NA	UP
**Gene16474**	**EGLN3**	NA	UP
**Gene3969**	MUT	NA	Down
**Gene21049**	FOXL2	Down	NA
**Gene23019**	PDP1	NA	UP
**Gene22580**	SERPINA7	Down	NA
**Gene11622**	TLX2	NA	Down
**Gene19027**	RHEBL1	NA	Down
**Gene6923**	SYP	NA	Down
**Gene466**	ECSCR	NA	UP
**Gene21566**	AQP11	NA	UP
**Gene18167**	PLEKHF2	UP	NA
**Gene12369**	RPL8	Down	NA
**Gene21551**	NONO	NA	Down
**Gene6891**	MPHOSPH6	NA	UP
**Gene6309**	KIF20B	NA	UP
**Gene16559**	PNO1	UP	NA
**Gene10955**	HCST	Down	NA
**Gene14033**	PDLIM3	NA	UP
**Gene5702**	HOXB7	NA	Down
**Gene21485**	ITM2A	Down	NA
**Gene17170**	43357	NA	UP
**Gene24124**	SMNDC1	UP	NA
**Gene22641**	MRPL20	NA	Down
**Gene7198**	INPP1	NA	Down
**Gene12913**	S100A1	NA	UP
**Gene6225**	FAM169A	NA	UP
**Gene17999**	TOP2B	NA	UP
**Gene16857**	PCTP	NA	Down
**Gene16714**	KLHL12	NA	UP
**Gene9820**	LMBRD1	NA	UP
**Gene8883**	ASPM	NA	UP
**Gene4484**	RGCC	NA	UP
**Gene23671**	CCL19	Down	NA
**Gene13542**	MED23	NA	UP
**Gene4051**	CDK5	NA	Down
**Gene17223**	LRRK2	NA	UP
**Gene14545**	FRG1	Down	NA
**Gene18760**	CPA3	NA	Down
**Gene11835**	C1QTNF4	NA	Down
**Gene24004**	LPL	UP	NA
**Gene24901**	UBQLN2	NA	Down
**Gene16856**	TMEM100	NA	UP
**Gene13441**	PYGO1	NA	Down
**Gene10329**	NDUFA9	NA	UP
**Gene1943**	TP53I3	NA	UP
**Gene10316**	BBOX1	NA	UP
**Gene17497**	IGSF6	UP	NA
**Gene20189**	UBE2C	Down	NA
**Gene22225**	MED17	NA	UP
**Gene21475**	HOXD12	UP	NA
**Gene9863**	DHX15	NA	UP
**Gene19229**	NFATC2IP	NA	Down
**Gene23893**	ABT1	NA	UP
**Gene14704**	TFB2M	NA	UP
**Gene8524**	LMOD2	NA	UP
**Gene12573**	PTMS	NA	Down
**Gene3328**	ERP44	NA	UP
**Gene15160**	APOC4	Down	Down
**Gene22228**	HEPHL1	NA	UP
**Gene21241**	SRPK3	NA	Down
**Gene14716**	SOX9	Down	NA
**Gene5821**	NKX2-5	UP	UP
**Gene2007**	PPRC1	NA	UP
**Gene12983**	PSMD10	NA	Down
**Gene5916**	OPALIN	NA	UP
**Gene12986**	ATP6V1C1	NA	UP
**Gene16429**	EMC4	UP	NA
**Gene19144**	MSMO1	NA	Down
**Gene16982**	SOX17	NA	Down
**Gene24358**	MAGEH1	Down	NA
**Gene19794**	SCGB3A1	NA	UP
**Gene11028**	WDR83	NA	Down
**Gene3820**	PGM2L1	NA	Down
**Gene11525**	FDX1L	NA	UP
**Gene10761**	GPR88	UP	NA
**Gene21912**	RPS7	NA	UP
**Gene7562**	GNAI1	NA	UP
**Gene8984**	SRD5A3	NA	Down
**Gene17461**	SLC25A20	NA	UP
**Gene13012**	CLUL1	NA	UP
**Gene13916**	TUBD1	NA	UP
**Gene20231**	EDA2R	NA	Down
**Gene13768**	ACVR2A	NA	UP
**Gene14993**	CLDN17	UP	NA
**Gene6350**	BTLA	NA	UP
**Gene703**	TMEM158	NA	Down
**Gene17409**	LUC7L3	NA	UP
**Gene21871**	ADAM2	NA	UP
**Gene14058**	HOXC6	NA	Down
**Gene24513**	PDK1	NA	UP
**Gene22089**	APCS	UP	NA
**Gene5079**	P2RY13	UP	NA
**Gene4646**	MIP	NA	UP
**Gene11440**	SPOCK3	NA	UP
**Gene6377**	OSTM1	NA	UP
**Gene11468**	TRA2A	NA	UP
**Gene9952**	TXLNG	NA	UP
**Gene24529**	EBAG9	NA	UP
**Gene22262**	FMO5	UP	NA
**Gene22794**	PINX1	NA	UP
**Gene3128**	HMGB1	Down	NA
**Gene5085**	SUCNR1	UP	NA
**Gene13898**	DECR1	NA	UP
**Gene9158**	CSN3	UP	NA
**Gene9671**	MMGT1	Down	NA

## Discussion

In the present study, we analyzed the methylation profile differences between the aged giant pandas suffering from cataract and healthy giant pandas and identified hundreds of DMGs in giant pandas with age-related cataract. Notably, we found no methylation differences on the genes (i.e., *GLB1*, *CDKN2A* and *CDKN2B*) highly correlated with aging[28–29], implying negligible effects of age on the DMGs. Further analysis showed that the genes with significant methylation differences between the case and control groups are indeed located on many biological processes related with cataract formation, such as base excision repair, p53 signaling pathway, and apoptosis[18, 30, 31]. Among them, p53 signaling pathway plays an important roles in the prevention of apoptosis of lens epithelial cells and cataractogenesis[31], the hypermethylation of genes (i.e., *TP53I3* and *CASP3*) on this pathway would like to downregulate the functions of p53-mediate signaling pathway and promote the development of cataract. In addition, other direct evidence comes from the certain genes that have been previously reported to be associated with cataract pathogenesis. For example, Glutathione S-Transferase Mu 3 (*GSTM3*, hypermethylated in giant pandas with cataract) was considered to prevent the age-related cataract by protecting the lens from oxidative stress and a decreased expression level of *GSTM3* was observed in the lens tissue of patients with age-related cataract, which correlated with the hypermethylation of *GSTM3* promoters[32].

Since DNA methylation is reversible and can be influenced by the external factors[33], the research on the appropriate epigenetic drugs based on the specific cataract-associated genes would be wildly used in prevention of age-related cataract development in giant pandas. In this study, we shed light on the methylation characteristics of giant panda suffering from cataract and provide a number of candidate epigenetic therapeutic targets for the prevention and treatment of cataract in the aged giant panda. Nevertheless, the small sample size and the lack of functional experiments limit the practical utility of the findings in this study. Therefore, further efforts are needed to address the issues as follows: 1) the validation of DMGs in a large giant panda population; 2) the influences of certain aberrant DNA methylation events on gene activity; 3) the key genes with major contributions to the cataract development in age giant pandas; 4) the molecular mechanisms of key genes in the pathogenesis of age-related cataract. In addition, we also observed that some giant pandas with cataract can be self-healing after their living environments were changed. The contribution of reversible epigenetic modifications (e.g., DNA methylation) caused by environmental stimulus to this phenomenon will be explored in our future studies.

## Conclusion

In short, we determined a number of DMGs that had potential roles in regulating the activity of cataract-related pathways, such as base excision repair, apoptosis, and p53 signaling pathway. Moreover, these findings were further supported by detailed genes with abnormal methylation pattern in giant pandas with cataract. For example, the *CASP3* gene encodes a cysteine-aspartic acid protease that served to as an apoptosis executor, and has been linked with cataract in rat[34–35]; *HMGB1* plays an important role in protecting the keratinocytes from ultraviolet radiation-induced cell death and is thus involved in cataract formation[36]. Overall, all the results argue for an important role of aberrant methylation changes in the development of cataract in aged giant pandas.

## Supporting information

S1 Table Methylation level of gene region. (XLSX)

S2 Table Genes associated with different methylation levels. (XLS)

S3 Table KEGG enrichment of genes related to different methylation levels. (XLSX)

S4 Table GO enrichment of genes related to different methylation levels. (XLSX)

S5 Table Cataract related genes have been reported. (XLS)
